# Effects of arsenic trioxide combined with platinum drugs in treatment of cervical cancer: A protocol for systematic review and meta-analysis of randomized controlled trials

**DOI:** 10.1097/MD.0000000000022950

**Published:** 2020-11-06

**Authors:** Yawen Zhang, Di Pan, Haishi Yang, Jiaxin Huang, Zeyang He, Haiying Li, Daocheng Li

**Affiliations:** The First Clinical College, Guangzhou University of Chinese Medicine, Guangzhou, Guangdong Province, P.R. China.

**Keywords:** arsenic trioxide (AS2O3), cervical cancer, meta-analysis, protocol, systematic review

## Abstract

**Introduction:**

Cervical cancer is the second largest tumor disease threatening female reproductive tract health. AS2O3 is a multi-directional and multi-target anti-cervical cancer drug. It can be combined with platinum drugs to treat cervical cancer. The literatures of AS2O3 combined with platinum drugs related to cervical cancer have shown inconsistent results, and there is currently no high quality of systematic review to evaluate the effects of AS2O3 combined with platinum drugs in cervical cancer patients.

**Methods and analysis:**

English and Chinese literature about AS2O3 combined with platinum drugs treatment for cervical cancer published before August 31, 2020 will be systematic searched in PubMed, Embase, Web of Science, Cochrane Library, Open Grey, Clinicaltrials.gov, Chinese Clinical Trial Registry, WANFANG, VIP Chinese Science and Technology Journal Database, CNKI, Chinese biomedical document service system (SinoMed). Only randomized controlled trials (RCTs) of patients with cervical cancer will be included. Literature screening, data extraction, and the assessment of risk of bias will be independently conducted by 2 reviewers, and the 3rd reviewer will be consulted if any different opinions existed. Clinical total effective rate, adverse events, SCCAg, CYFRA21-1, quality of life, and immune function will be evaluated. Systematic review and meta-analysis will be produced by RevMan 5.3 and Stata 14.0. This protocol reported in accordance with the Preferred Reporting Items for Systematic Review and Meta-analysis Protocols (PRISMA-P) statement, and we will report the systematic review by following the PRISMA statement.

**Results:**

The current study is a protocol for systematic review and meta-analysis without results, and data analysis will be carried out after the protocol. We will share our findings in the fourth quarter of 2021.

**Conclusion:**

Efficacy and safety of AS2O3 combined with platinum drugs in the treatment of cervical cancer will be assessed. The results will be published in a public issue journal to provide evidence-based medical evidence for Obstetrician and Gynecologists to make clinical decisions.

**Ethics and dissemination:**

Ethical approval is not required as the review is a secondary study based on published literature. The results of the study will be published in peer-reviewed publications and disseminated electronically or in print.

**Protocol registration number:**

INPLASY202080130.

## 1 Introduction

Cervical cancer is the most common malignant tumor of the female reproductive tract and the second largest tumor disease threatening female reproductive health.^[1]^ There are about 500,000 new cases of cervical cancer each year, of which >80% are in developing countries, and >260,000 women die from cervical cancer each year, mainly in low- and middle-income countries.^[2]^ For patients with early cervical cancer, the NCCN guidelines recommend radical hysterectomy for cervical cancer and determine whether adjuvant treatment is needed according to the postoperative pathological results. The 5-year survival rate of patients may be as high as 80% to 90%.^[3,4]^ Although the prevention and treatment of cervical cancer have been continuously improved, in developing countries, two-thirds of patients are still treated at the end of the local stage, and the 5-year survival rate is only 30% to 80%.^[5]^ For patients with IB, IIA, and IIB stages and above, the recommendations of the guidelines are different and are based primarily on platinum-based drugs. However, some patients with carboplatin resistance have poor survival prognosis. Therefore, we need to explore more effective chemotherapy drugs.

Arsenic trioxide (As2O3) is an effective ingredient of traditional Chinese medicine arsenic, which has been used in the treatment of solid tumors in recent years.^[6]^ It is also a multi-directional and multi-target anti-tumor drug, which can interfere with different stages of tumor to achieve the purpose of anti-tumor.^[7–10]^ In recent years, cell and animal experiments showed that As2O3 had significant anti-tumor effect on cervical cancer. Some clinical trials also showed that it has significant effects in the treatment of cervical cancer. However, there are still inconsistent results in the clinical benefits whether As2O3 combined with platinum drugs have a better efficacy in the treatment of cervical cancer. And randomized controlled trials (RCTs) provide the most reliable evidence for medical intervention,^[11]^ and the quality of evidence is higher than that of observational studies.^[12]^ Since there is currently no systematic review of AS2O3 combined with platinum drugs for treatment of cervical cancer based on RCTs. Therefore, it is necessary to carry out a systematic review and meta-analysis to fully evaluate the efficacy and safety of As2O3 combined with platinum drugs in the treatment of cervical cancer.

## 2 Materials and methods

This protocol refers to the statement of Preferred Reporting Items for Systematic Review and Meta-analysis Protocols (PRISMA-P).^[13,14]^ And we will report the systematic review by following the PRISMA statement. This protocol has been registered with the International Platform of Registered Systematic Review and Meta-analysis Protocols (registration number: INPLASY202080130) which could be available on https://inplasy.com/.

### 2.1 Eligibility criteria

We will include studies according to the criteria outlined below.

#### 2.1.1 Study designs

This study will include only RCTs. Other studies such as observational studies, retrospective analyses, self-controlled trials, patient series, case reports, reviews, animal studies, and laboratory in vitro studies will be excluded.

#### 2.1.2 Participants

Patients with cervical cancer diagnosed pathologically; the staging standard refers to FIGO in 2008: IB2, IIA, IIB; 20 to 60 years old; blood analysis before treatment, normal liver, and kidney function; no evidence of distant metastasis; no serious heart, liver, kidney, and blood system, and other important organ diseases. The patient has signed an informed consent form.

#### 2.1.3 Interventions

Radical hysterectomy for cervical cancer after 2 courses of chemotherapy (AS2O3 combined with platinum drugs).

#### 2.1.4 Comparisons

Radical hysterectomy for cervical cancer after 2 courses of chemotherapy (TP chemotherapy regimen).

#### 2.1.5 Outcomes

The potential outcomes of our interest contain the following:

##### 2.1.5.1 Primary outcomes

Clinical total effective rate and adverse reactions: according to the World Health Organization (WHO) solid tumor efficacy evaluation criteria, it is divided into complete response (CR), partial response (PR), stable disease (SD), and progressive disease (PD); total clinical effective rate = (number of CR cases + number of PR cases)/total number of cases × 100%, disease control rate = (number of CR cases + number of PR cases + number of SD cases)/total number of cases × 100%.^[15]^ Adopt the WHO “Acute and Subacute Toxicity Classification Standards of Anticancer Drugs,” divide the adverse reactions into 0 to IV grades, observe the occurrence of gastrointestinal reactions (grade 0–IV) and bone marrow suppression (grade 0–IV); at the same time pay attention to the occurrence of complications.

##### 2.1.5.2 Secondary outcomes

Cervical cancer tumor markers (SCCAg/CYFRA21–1), quality of life, and immune function.

### 2.2 Search methods

#### 2.2.1 Information sources

PubMed, Science Citation Index, Embase (Ovid) database, the Cochrane Library, and 4 Chinese databases (the China National Knowledge Infrastructure, the China Biology Medicine disc, the China Science and Technology Journal Database, and the Wan fang Database) will be searched from database inception to August 31, 2020. ClinicalTrials.gov and the Chinese Clinical Trial Registry Platform will be searched for ongoing or recently completed trials. Besides, we will scan the reference lists of included studies or relevant reviews to identify additional eligible studies, while the papers and unpublished reports will be hand-searched to ensure more complete coverage of the topic.

#### 2.2.2 Search strategies

Subject heading, lower words, entry terms, and free words search will be used in PubMed, Embase, and Cochrane library. Cochrane library search will be restricted by using “search word variations.” Topic search will be used in Web of Science. Free words will be searched within title, abstract, keywords in Cochrane library, Embase and within title, and abstract in PubMed. Chinese database search: CNKI will be restricted by using “topic” field; WANFANG and VIP will be limited by “title or keyword” filed; SinoMed will be searched by using subject words search plus synonym retrieval.

Search terms include: “cervical cancer” or “carcinoma of cervix” or “cervical carcinoma” or “carcinoma cervicis” and “Arsenic Trioxide” or “Arsenite” or “white arsenic” or “arsenous oxide” and “platinum drugs.” Chinese search will use the Chinese form of the above terms. The example of specific search for PubMed is shown in Table 1.

**Table 1:** This table presents the initial draft of the search strategy with PubMed as an example.

Number	Search terms
#1	cervical cancer [Mesh]
#2	carcinoma of cervix[Mesh] OR carcinoma of cervix [All Fields] OR carcinoma cervicis [All Fields] OR cervical carcinoma [All Fields]
#3	Arsenic Trioxide[Mesh] OR Arsenite [All Fields] OR arsenous oxide [All Fields]white arsenic [All Fields]
#4	platinum drugs
#5	#1 OR #2
#6	#3 AND #4
#7	#5 AND #6

### 2.3 Data collection

#### 2.3.1 Selection of studies

**Figure 1:**
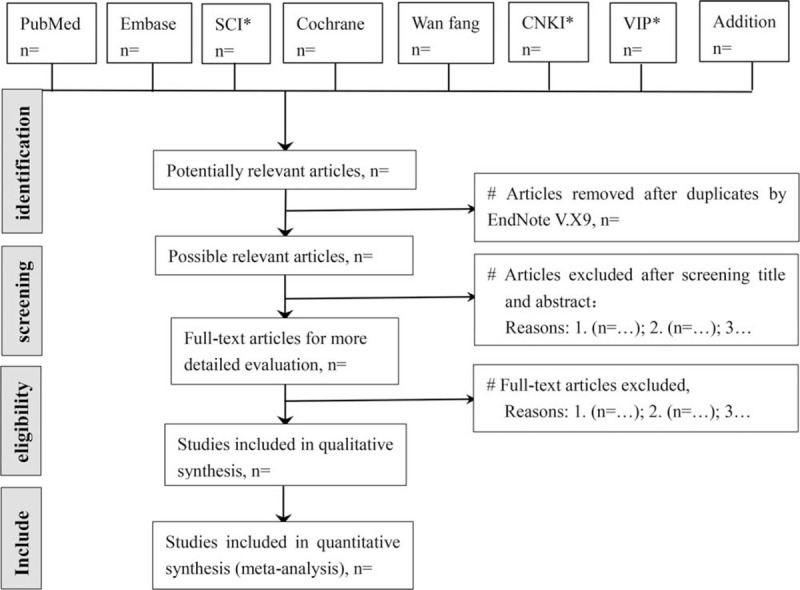
Study selection flow chart. CBM = China Biology Medicine disc, CNKI = China National Knowledge Infrastructure, SCI = Science Citation Index, VIP = China Science and Technology Journal Database.

According to pre-defined eligibility criteria, the screening will be carried out in duplicate by 2 independent reviewers (YZ and DP) at each stage of the review. Studies will be removed if they don’t meet the inclusion criteria obviously. If the studies appear to meet the inclusion criteria or there is any uncertainty based on the information provided in the title and abstract, full texts will be obtained for further assessment. When necessary, we will contact the author for more details of the study to solve questions about eligibility. Disagreements will be resolved by discussion or consulting expert (DL) for arbitration. The number and reasons for excluding trials will be recorded in detail. A flow diagram of the study selection is shown in Fig. 1.

#### 2.3.2 Data extraction

Data extraction for eligible studies will be performed independently by 2 reviewers (YZ and DP) using a pre-designed standardized form. We will provide guidance and interpretation for the contents of the extraction form before data extraction. The detailed data extraction form will mainly consist of basic information, population characteristics, methodological description, intervention characteristics, outcome data, conclusion, and follow-up assessment. We will contact the original researchers for missing data. The third reviewer (HY) will be responsible for checking the data extracted by the 2 reviewers. Inconsistencies will be resolved by discussion, and consulting the superior expert (DL) to facilitate the decision when a disagreement persisting.

### 2.4 Assessment of risk of bias

The methodological quality of individual studies will be judged following the criteria from the Cochrane Handbook for Systematic Reviews of Interventions Version 5.3.0.^[16]^ The judgments of all included studies will be made independently by 2 reviewers (YZ and DP), and we will conduct training of reviewers and calibration exercises before the start of the review to ensure consistency between reviewers. There are 7 domains, each of which will be rated as “yes” (indicating a low risk of bias), “no” (indicating a high risk of bias), or “unclear” (indicating either an uncertainty for bias or lack of information). The original study investigators will be contacted if any uncertainty exists. We plan to compute graphic representations of potential bias within and across studies using Review Manager 5.3. Those with inconsistent opinions will be resolved through negotiation or consult the superior expert (DL) to reach a consensus. Overall, the following aspects will be considered:

(1) Appropriate generation of random allocation sequence (selection bias);
(2) Concealment of the allocation sequence (selection bias);
(3) Blinding of participants and healthcare providers (performance bias);
(4) Blinding of data collectors and outcome adjudicators (detection bias);
(5) Incomplete outcome data such as dropouts and withdrawals (attrition bias);
(6) Selective outcome reporting (publication or dissemination bias);
(7) Other bias (such as sponsorship bias).

### 2.5 Data analysis

#### 2.5.1 Data synthesis and meta-analysis

We will perform a systematic narrative synthesis to summarize and explain the characteristics and findings of the included studies and provide this information in the text and tables. Review Manager 5.3 provided by the Cochrane Collaboration will be used for the meta-analysis (if feasible), and the random-effects model will be chosen to combine all summary outcome measures. If a meta-analysis is impossible, the results of clinical trial comparisons will be analyzed descriptively. Dichotomous outcomes (e.g., effective and ineffective) will be determined by relative risk (RR) with 95% confidence interval (CI), while continuous data will be analyzed using weighted mean difference (if measurement methods are consistent) or standardized mean difference (if measurement methods are different).

#### 2.5.2 Dealing with missing data

When there are missing data, we will contact the study authors via email to obtain detailed accurate data. If the missing data are not available finally, we will carefully estimate the important numerical data, for example using an interpolation method. Moreover, the potential impact of missing data on the overall results of the study will be assessed using sensitivity analysis. It is possible to include multi-arm trials, we will combine the relevant groups into a single group according to the formula provided in the Cochrane handbook 5.3.0.^[16]^

#### 2.5.3 Assessment of heterogeneity and publication bias

Heterogeneity of each outcome measure will be tested using the chi-square test and *I*^2^ statistic.^[17]^ If there is significant heterogeneity among the trials (*I*^2^ ≥ 50% or *P* < .1), we will try to explain the source of heterogeneity through subgroup analysis or sensitivity analysis. And we should not perform a meta-analysis if heterogeneity is substantial, a narrative qualitative summary will be done instead. Funnel plot will be used to reveal potential publication bias if over 10 studies are available.^[18]^

#### 2.5.4 Subgroup analysis and sensitivity analysis

Subgroup analysis will be performed according to age, ethnicity, FIGO 2008 stage, and Histological grade and type of cervical cancer. We will use sensitivity analysis to test the stability and reliability of meta-analysis. It will be conducted by 2 methods: eliminating each study one by one; using random-effect model (DerSimonian and Laird method) to test the results after using the fixed effect model.^[19,20]^

### 2.6 Grading the quality of evidence

The quality of evidence in the systematic review will be judged by the GRADE tool.^[21]^ It is based on 5 key domains: risk of bias, consistency, directness, precision, and publication bias. The evidence levels for each outcome will be adjudicated as high quality, moderate quality, low quality, and very low quality.^[22]^ RCTs with low risk of bias are considered high-quality evidence that could provide a direct and precise reference for clinical application.

### 2.7 Reporting of the review

The methodological quality of the systematic review and meta-analysis to be completed next will be standardized by each item of the AMSTAR-2 tool.^[23]^ And the results will be reported following the Preferred Reporting Items for Systematic Reviews and Meta-Analysis (PRISMA) statement published in 2009.^[24]^

## 3 Discussion

Cervical cancer seriously affects women's health. In developing countries, its morbidity and mortality are significantly higher than those in Western developed countries, because most patients with cervical cancer are in advanced stage in developing countries, and the prognosis is poor.^[5,25–27]^ Combination chemotherapy based on platinum drugs are often used clinically, but their adverse reactions are large and drug resistance is easy to develop. In recent years, many cell and animal experiments have shown that arsenic trioxide can have anti-tumor effects through multiple mechanisms, such as: inducing tumor cell differentiation, apoptosis, autophagy; inhibiting cancer cell growth and proliferation, tumor angiogenesis, invasion and metastasis; reversing multi-drug resistance, etc. And arsenic trioxide has significant clinical effects in the treatment of hematological malignancies and various solid malignancies. In addition, in developing countries, the anti-cervical cancer effect of As2O3 has the unique advantages of high efficiency and multi-targets. It can be combined with other chemotherapeutics to minimize adverse reactions and also exert a synergistic anti-cancer effect.^[28–30]^

This systematic review has the following limitations: first, as we are not good at other languages, the literatures we searched are limited to Chinese and English, which will cause certain bias. Second, there may be a limited number and sample size of RCT for treating cervical cancer, the quality of evidence provided may not be high. Third, the limitation of sample size also leads to the instability of conclusion reliability. Therefore, we hope that there will be more large-scale, multicenter, high-quality RCTs in the future to provide high-quality evidence.

## Author contributions

**Conceptualization:** Yawen Zhang.

**Data curation:** Yawen Zhang, Di Pan, Haishi Yang.

**Formal analysis:** Di Pan, Jiaxin Huang, Haishi Yang.

**Funding acquisition:** Yawen Zhang.

**Methodology:** Yawen Zhang, Di Pan.

**Project administration:** Yawen Zhang, Daocheng Li.

**Software:** Di Pan, Haishi Yang, Jiaxin Huang, Zeyang He.

**Supervision:** Daocheng Li.

**Validation:** Di Pan, Haishi Yang, Haiying Li.

**Writing – original draft:** Yawen Zhang, Di Pan, Haishi Yang, Jiaxin Huang.

**Writing – review & editing:** Yawen Zhang, Di Pan, Daocheng Li.
